# Structures, Interactions and Activity of the N-Terminal Truncated Variants of Antimicrobial Peptide Thanatin

**DOI:** 10.3390/antibiotics13010074

**Published:** 2024-01-12

**Authors:** Swaleeha Jaan Abdullah, Yuguang Mu, Surajit Bhattacharjya

**Affiliations:** School of Biological Sciences, Nanyang Technological University, Singapore 637551, Singapore; swaleeha002@e.ntu.edu.sg (S.J.A.); ygmu@ntu.edu.sg (Y.M.)

**Keywords:** thanatin, antibiotic resistance, antimicrobial peptides, NMR

## Abstract

Gram-negative bacteria are intrinsically more resistant to many frontline antibiotics, which is attributed to the permeability barrier of the outer membrane, drug efflux pumps and porins. Consequently, discovery of new small molecules antibiotics to kill drug-resistant Gram-negative bacteria presents a significant challenge. Thanatin, a 21-residue insect-derived antimicrobial peptide, is known for its potent activity against Enterobacter Gram-negative bacteria, including drug-resistant strains. Here, we investigated a 15-residue N-terminal truncated analog PM15 (P^1^IIYCNRRTGKCQRM^15^) of thanatin to determine modes of action and antibacterial activity. PM15 and the P^1^ to Y and A substituted variants PM15Y and PM15A delineated interactions and permeabilization of the LPS–outer membrane. In antibacterial assays, PM15 and the analogs showed growth inhibition of strains of Gram-negative bacteria that is largely dependent on the composition of the culture media. Atomic-resolution structures of PM15 and PM15Y in free solution and in complex with LPS micelle exhibited persistent β-hairpin structures similar to native thanatin. However, in complex with LPS, the structures of peptides are more compact, with extensive packing interactions among residues across the two anti-parallel strands of the β-hairpin. The docked complex of PM15/LPS revealed a parallel orientation of the peptide that may be sustained by potential ionic and van der Waals interactions with the lipid A moiety of LPS. Further, PM15 and PM15Y bind to LptA_m_, a monomeric functional variant of LptA, the periplasmic component of the seven-protein (A-G) complex involved in LPS transport. Taken together, the structures, target interactions and antibacterial effect of PM15 presented in the current study could be useful in designing thanatin-based peptide analogs.

## 1. Introduction

Antibiotics are still the best drugs for eradicating infections caused by pathogenic bacteria. However, usage of antibiotics is severely limited against Gram-negative bacteria compared to Gram-positive ones. A wide range of potent antibiotics, such as vancomycin, novobiocin and azithromycin, are known to be clinically ineffective in treating infections caused by Gram-negative bacteria [1,2,3]. These antibiotics are excluded by the permeability barrier of the LPS–outer membrane and also cannot use the porin channel, molecular weight (>600 Da), to enter into cellular milieu [4,5,6]. At present, the rapid increase in antibiotic resistance among pathogenic bacteria has made it extremely challenging to develop effective antibiotics for Gram-negative bacteria [7,8,9,10]. Analyses of global AMR data for the year 2019 have estimated that bacterial AMR was responsible for around 4.9 million deaths [7]. A previous report from O’Neill and collaborators indicated that 10 million annual deaths might occur globally due to AMR [8]. The leading pathogens that cause most AMR-related fatalities are Gram-negative bacteria, namely, drug- or multi-drug-resistant strains of *E. coli*, *K. pneumoniae*, *A. baumannii* and *P. aeruginosa* [7,8,9,10]. At present, infections caused by the resistant strains of Gram-negative pathogens are extremely hard to treat in intensive care units [7,8,9,10]. Strains of *K. pneumonia, A. baumannii* and *P. aeruginosa* have been detected which are resistant to carbapenem, the last-resort beta-lactam antibiotic [11,12]. Extremely drug-resistant Gram-negative bacteria are often treated with nephrotoxic cyclic peptides colistin or polymyxin B [13,14]. 

Antimicrobial peptides (AMPs) are found in all life forms and constitute the first line of defense as a part of innate immunity in higher organisms, including humans [15,16,17,18]. As a mode of action, a significant number of cationic AMPs disrupt membrane structures including the LPS–outer membrane of Gram-negative bacteria [19,20,21,22]. Thanatin, a 21-residue antimicrobial peptide, was originally identified from the hemolymph of the spined soldier bug, or *Podisus maculiventris* [23]. Recent studies have discovered and characterized thanatin orthologous AMPs from other species of insects [24,25]. Thanatin exerts a broad spectrum of antimicrobial activity in relation to Gram-negative and Gram-positive bacteria and against several strains of fungi [23,26,27,28,29,30]. Interestingly, thanatin has been found to be extremely potent in selectively killing Gram-negative bacteria belonging to the family of Enterobacteriaceae, including drug-resistant strains [26,28,30,31]. In vivo studies demonstrated a promising activity profile of the thanatin peptide in clearing bacterial load from model animals [29,30,31]. As a mode of action against Gram-negative bacteria, thanatin interacted with the LPS–outer membrane, facilitating the permeabilizing of the OM barrier [27,28,32]. In addition, thanatin inhibits outer-membrane biogenesis by disrupting protein complexes (LptA and LptD) involved in the LPS transport process [33,34,35]. Due to high antibacterial activity and a unique mode of action, native thanatin and its functional shorter analogs possess a high potential for the development of antibiotics to mitigate drug-resistant Gram-negative bacterial pathogens [26,28,31]. In this work, we report an in-depth characterization of a 15-residue thanatin peptide, or PM15, and its analogs in terms of atomic-resolution structures, mode of action and antibacterial activity. Mechanistic insights obtained from this study could be useful in developing the modality of thanatin-based antibiotics. 

## 2. Results

### 2.1. Thanatin Peptide Fragments and Antibacterial Activity 

Previous studies showed that the N-terminal deletion fragments of thanatin can retain antibacterial activity [23,31,36]. In particular, a 16-residue fragment (VPIIYCNRRTGKCQRM) of thanatin demonstrated antibacterial activity [23,31,36], whereas a 14-residue (IIYCNRRTGKCQRM) fragment was found to be largely inactive [23,36]. Further, to understand residue length-dependent antibacterial activity, here, we investigated a 15-residue N-terminal peptide fragment, or PM15 (PIIYCNRRTGKCQRM), of thanatin. We also examined two analog peptides, PM15Y and PM15A, to replace residue Pro. The analog peptides were designed based on the premise that the inclusion of residue Tyr in PM15Y peptide might enhance membrane interactions, whereas PM15A would act as a control. MIC values of the three peptides, PM15, PM15Y and PM15A, were determined against representative strains of Gram-negative and Gram-positive bacteria in two MH broth media (Table 1). PM15 and PM15A peptides demonstrated growth inhibition of the bacterial strains with an MIC value ranging from 4 to 8 µM in an aged, about 5 years, MH broth medium. PM15Y peptide was able to inhibit growth of all the bacterial strains except for *S. enterica*. Also, high MIC > 16 µM could be seen for the peptides against *A. baumannii*. However, surprisingly, no bacterial growth inhibition was observed for all three peptides in a new MH broth (Sigma-Millipore, St Louis, MO, USA). These observations are not immediately clear to us. We suspect that the composition of the media might have influenced the antibacterial activity of the PM15 peptides. The new MH broth showed intense color, potentially indicating varied compositions. To better understand the antibacterial performance of the PM15 peptides, structural, biophysical and target interaction analyses were carried out.

### 2.2. Surface Charge Neutralization, Outer-Membrane Permeabilization and LPS Interactions

**Figure 1:**
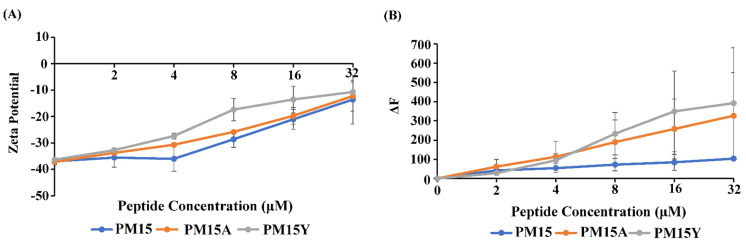
Bacterial surface neutralization and LPS–outer membrane permeabilization by PM15, PM15Y and PM15A peptides. (**A**) Changes in zeta potential of *E. coli* cells as a function of concentrations of PM15, PM15Y and PM15A peptides. (**B**) Changes in fluorescence intensity at the emission maxima of NPN upon addition of PM15, PM15Y and PM15A peptides.

Figure 1A shows zeta potential values of *E. coli* cells as function of concentrations of PM15, PM15Y and PM15A peptides. Gram-negative bacteria display a large negative zeta potential due to the presence of anionic phosphates and carboxylate groups of the LPS–outer membrane. Binding of cationic AMPs would cause a neutralization of the bacterial surface charge that may be correlated with OM permeabilization and antibacterial activity [37,38,39]. As seen in the figure, additions of PM15 and analog peptides, PM15Y and PM15A, in *E. coli* cell solutions caused an increase in zeta potential in a dose-dependent manner (Figure 1A). Apparently, at lower doses, 4–16 µM, PM15Y peptide delineates a higher surface charge neutralization compared to PM15 and PM15A peptides. 

The ability of the peptides to permeabilize the integrity of the LPS–outer membrane has been assessed by fluorescence of hydrophobic NPN probe using *E. coli* cells. NPN is excluded by the bacterial membrane, exhibiting poor fluorescence emission. NPN fluorescence can significantly increase within the non-polar milieu of bacterial membrane in the presence of membrane-perturbing agents, including AMPs [40,41]. Figure 1B presents changes (ΔF) in NPN fluorescence emission intensity upon inclusion of thanatin-derived peptides in *E. coli* cell solutions. As seen in the figure, all three peptides caused an increase in NPN fluorescence in a concentration-dependent way, indicating a probable disruption of the LPS–outer membrane. Notably, PM15Y and PM15A peptides yielded a higher increase in NPN fluorescence compared to the parent PM15 peptide (Figure 1B). These observations may suggest a superior membrane permeabilization by the analog peptides. 

**Figure 2:**
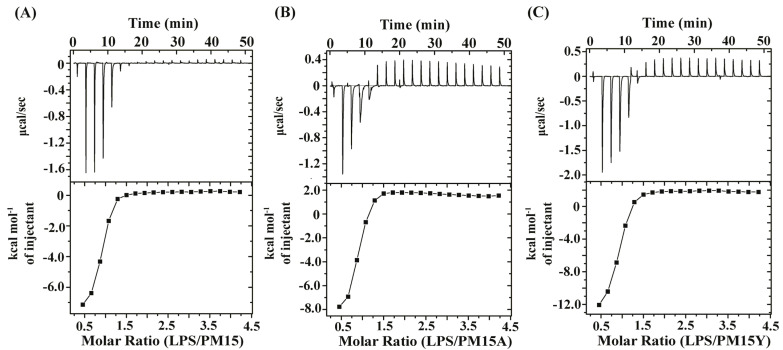
Thermodynamics of interactions of LPS–outer membrane with PM15, PM15Y and PM15A peptides revealed by ITC experiments. (**A**–**C**) ITC measurements of 1 mM of peptides PM15 (**A**), PM15Y (**B**) and PM15A (**C**) titrated into 50 μM *E. coli* LPS in 10 mM sodium phosphate, pH 7.0 at 37 °C. The upper panel of the ITC thermogram shows the heat exchange peaks plotted as power (μcal s^−1^) against time (min). The lower panel shows the resulting integrated heat exchange peaks of the binding. LPS-peptide binding parameters are listed in Table 2.

Next, we examined the thermodynamics of binding interactions of the peptides with the LPS–outer membrane by using ITC experiments (Figure 2A–C). All three peptides interacted with LPS and exhibited an exothermic binding, as evident from the negative values of heat-exchange processes (Figure 2A–C, upper panels). ITC thermograms were analyzed by estimating binding and thermodynamic parameters (Table 2). As seen in Table 2, peptide/LPS interactions are driven by favorable changes of enthalpy to the ΔG values. In addition, the analog peptides PM15Y and PM15A displayed relatively high-affinity interactions with LPS compared to the parent peptide PM15 (Table 2). 

### 2.3. NMR Analyses of PM15 and PM15Y Peptides 

**Figure 3:**
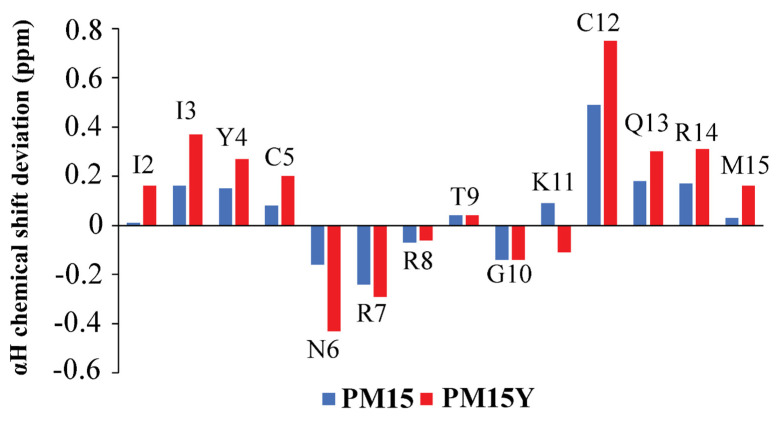
NMR secondary chemical shift (ppm) of PM15 and PM15Y peptides. Plot shows deviation of ^α^H chemical shifts of individual amino acid of PM15 and PM15Y peptides from the random coil chemical shift. A positive deviation in secondary chemical shift indicates β-strand conformation.

**Figure 4:**
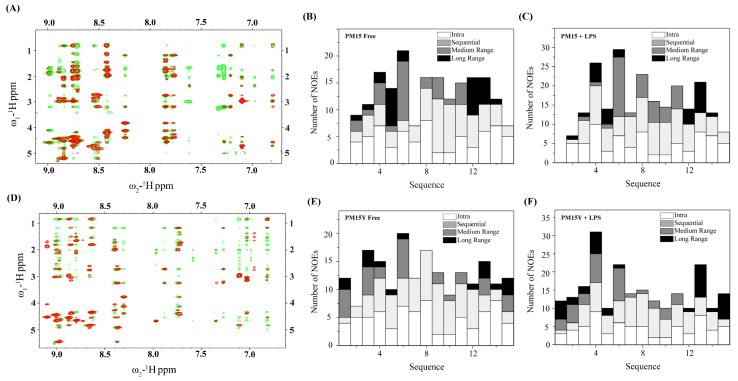
NMR studies of PM15 and PM15Y peptides in free solution and in complex with LPS micelle. (**A**) Overlay of the ^1^H-^1^H two-dimensional NOESY spectra of PM15 peptide showing NOE correlations among the proton resonances at the low-field, along the ω_2_ dimension (in ppm), with the up-field, along ω_1_ dimension (in ppm), in free solution (red contour) and in complex with LPS micelle (green contour). (**B**,**C**) Bar diagrams showing type and number of NOEs observed for PM15 peptide in (**B**) free solution and (**C**) complex of LPS micelle. (**D**) Overlay of the ^1^H-^1^H two-dimensional NOESY spectra of PM15Y peptide showing NOE correlations among proton resonances at the low-field, along the ω_2_ dimension (in ppm), with the up-field, along ω_1_ dimension (in ppm), in free solution (red contour) and in complex with LPS micelle (green contour). (**E**,**F**) Bar diagrams showing type and number of NOEs observed for PM15Y peptide in (**E**) free solution and (**F**) complex of LPS micelle.

Sequence-specific resonance assignments of PM15 and PM15Y peptides were achieved by combined analysis of TOCSY and NOESY spectra. Positive deviation of secondary chemical shifts of ^α^H of residues of PM15 and PM15Y delineated β-sheet conformations of residues I3, Y4, C5, K11, C12, Q13 and R14, whereas, intervening residues N6, R7, R8 and G10 experienced a negative deviation of ^α^H secondary chemical shifts (Figure 3). The secondary chemical shift pattern typically indicates β-hairpin conformations of the peptides [42]. Two-dimensional ^1^H-^1^H NOESY spectra of peptides PM15 and PM15Y delineated more and intense NOEs in complex with LPS micelle compared to the free peptide (Figure 4A,D). Table 3 and Table 4 list long-range NOEs detected for PM15 and PM15Y in free solution and in complex with LPS micelle, respectively. 

Bar diagrams summarize the number of NOEs in terms of categories assigned for each residue of PM15 (Figure 4B,C) and PM15Y (Figure 4E,F) in free solution and in complex with LPS micelle. Notably, analyses of the NOESY spectra of the peptides in complex with LPS micelle revealed many more long-range NOEs compared to those in free solution. These observations indicate plausible structural reorganization of the peptides upon binding to the LPS–outer membrane. Interestingly, PM15Y peptide demonstrated long-range NOEs involving the aromatic sidechain protons of residue Y1 with the sidechain protons of residue M15 (Figure 4E,F and Table 4). Such long-range NOEs between residues P1 and M15 of PM15 were not detected.

### 2.4. Atomic-Resolution Structures of PM15 and PM15Y in Free Solution and in Complex with LPS Micelle

**Figure 5:**
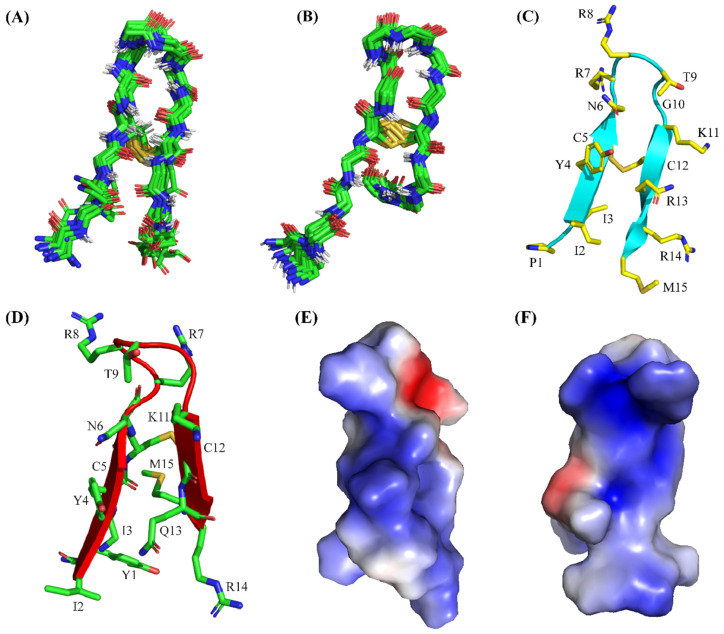
NMR-derived structures of PM15 and PM15Y peptides in free solution. Backbone superpositions of twenty low-energy structures of (**A**) PM15 and (**B**) PM15Y peptides obtained from CYANA. Ribbon representation of the three-dimensional structure of peptides (**C**) PM15 and (**D**) PM15Y showing backbone and sidechain orientations. Electrostatic potential diagrams of the peptides (**E**) PM15 and (**F**) PM15Y showing patches of cationic surfaces (in blue) in the β-hairpin structures.

NOE-driven distance and backbone dihedral angle (φ,ψ) constraints are utilized to solve three-dimensional structures of PM15 and PM15Y peptides by CYANA [43]. Figure 5 shows backbone (^α^C, N and C′) superpositions of twenty low-energy structures of PM15 (Figure 5A) and PM15Y (Figure 5B) in free solutions. Table 5 summarizes constraints and structural statistics. As seen in the table, canonical β-hairpin structures are persistent, akin to full-length thanatin, for PM15 (Figure 5C) and PM15Y (Figure 5D) peptides. 

Notably, NMR-derived structures of 21-residue thanatin demonstrated folded β-hairpin structures only for residues I8-M21, whereas the first seven N-terminal residues (G^1^SKKPVP^7^) belonging to the native peptide assumed flexible conformations in free solution [27,32,44,45]. Electrostatics potential shows discrete patches of cationic surfaces of the β-hairpin structures of PM15 (Figure 5E) and PM15Y (Figure 5F). 

**Figure 6:**
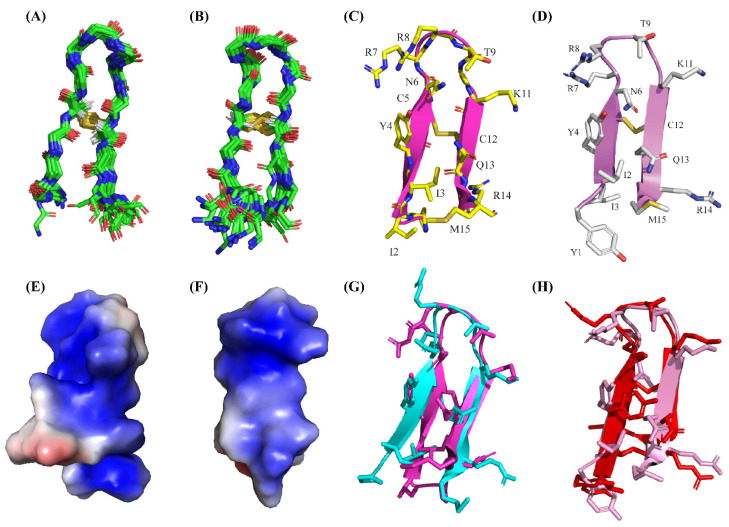
NMR-derived structures of PM15 and PM15Y peptides in complex with LPS micelles. Backbone superpositions of twenty low energy structures of peptides (**A**) PM15 and (**B**) PM15Y in complex with LPS obtained from CYANA. Ribbon representation of the three-dimensional structure of the peptides (**C**) PM15 and (**D**) PM15Y showing backbone and sidechain dispositions. Electrostatic potential diagrams of the peptides (**E**) PM15 and (**F**) PM15Y showing organized cationic surfaces (in blue) in the β-hairpin structures in complex with LPS micelle. Ribbon representation of the structural superpositions of the peptides (**G**) PM15 and (**H**) PM15Y in free solution and in complex with LPS micelle.

Backbone superpositions of twenty low-energy structures are presented for PM15 (Figure 6A) and PM15Y (Figure 6B) in complex with LPS micelle. Noticeably, the well-defined structural ensemble of PM15 in complex LPS micelle can be realized as estimated RMSD values, and backbone and all heavy atoms are lower compared to those of free peptide (Table 4). However, the RMSDs of the structural ensembles of PM15Y peptide appear to be somewhat higher in complex with LPS micelle compared to its free counterpart (Table 5). The β-hairpin structures of PM15 (Figure 6C) and PM15Y (Figure 6D) are defined in complex of LPS micelle. Electrostatic surface potential of PM15 and PM15Y in complex with LPS micelle delineated a well-organized cationic face of the molecule (Figure 6E,F). 

However, significant differences are notable between the β-hairpin structures of PM15 peptide in free solution and in complex of LPS (Figure 6G). Backbone superpositions of the two structures revealed an RMSD of 1.67 Å. In particular, residues at the N-termini of the β-hairpin have deviated more compared to the residues of the C-termini and loop (Figure 6G). Backbone superposition of the β-hairpin structures of PM15Y peptide also demonstrated a significant variation with an estimated RMSD of 2.67 Å (Figure 6H). 

### 2.5. Thermodynamics of Interactions of PM15 and PM15Y Peptides with LptA_m_

**Figure 7:**
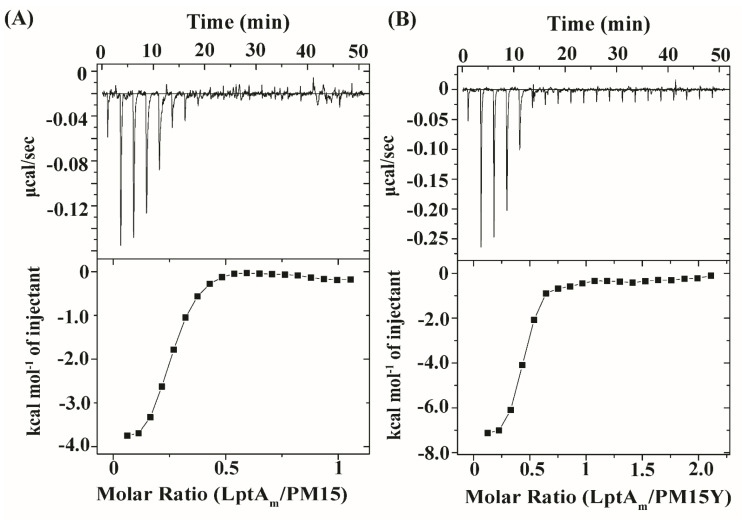
Binding interactions of PM15, PM15Y peptides with protein LptA_m_ by ITC experiments. ITC measurements of 250 μM of peptides (**A**) PM15 and (**B**) PM15Y titrated into 25 μM LptA_m_ in 50 mM sodium phosphate buffer, 150 mM NaCl, pH 7.0 at 25 °C. The upper panel of the ITC data shows the heat exchange peaks plotted as power (μcal s^−1^) against time (min). The lower panel shows the resulting integrated heat exchange peaks of the binding. LPS–peptide binding parameters are listed in Table 6.

Figure 7 shows ITC studies of PM15 (Figure 7A) and PM15Y (Figure 7B) with LptA_m_ protein of *E. coli*. As seen in the figure, both peptides demonstrated high-affinity binding with the target protein, as indicated by the exothermic (downward peaks) heat-exchange process in a saturable manner (Figure 7). The apparent dissociation constant (K_d_) values of LptA_m_ binding are determined to be 0.6 µM and 0.4 µM for PM15 and PM15Y peptides, respectively. Thermodynamic parameters of LptA_m_ binding with the peptides are provided in Table 6. 

## 3. Discussion 

Thanatin and its orthologs are single disulfide-bonded short AMPs involved in insect host defense [23,24,25,26]. The novel mode of action of thanatin is attractive for developing peptide antibiotics to kill drug-resistant Gram-negative pathogens [27,28,31,46,47,48]. Due to high cationicity, thanatin interacts with the LPS–outer membrane of Gram-negative bacteria to disrupt the permeability barrier [27,28,32]. The process is believed to help in gaining access to bacterial periplasmic space where thanatin binds to the protein components, LptA and LptD, involved in LPS transport [33,34,35]. Truncated or minimized analogs of thanatin could be useful in designing a peptide library for efficient selection of antibiotics [31,46,48]. 

Notably, N-terminally deleted 16-residue thanatin can demonstrate potent antibacterial activity, retaining a mode of action akin to native thanatin [23,26,31,36]. However, deletion of two more residues, that is, 14-residue thanatin, yielded an inactive peptide, indicating a probable delicate balance of amino acid residues with antibacterial activity and mode of action [23,36]. In this study, we have examined 15-residue thanatin-derived peptide, or PM15, and two analogs, PM15Y and PM15A. PM15 and analog peptides are able to exert antibacterial activity against Gram-negative and Gram-positive strains (Table 1). However, antibacterial activity appears to be significantly attenuated in a rich medium. Nonetheless, PM15 and analog peptides could demonstrate high-affinity LPS binding and permeabilization of the LPS–outer membrane. Peptide–LPS binding is enthalpically driven, suggesting potential involvement of ionic and/or hydrogen bonding in the complex formation (Table 2). Similar binding energetics are reported for the native thanatin with the LPS–outer membrane [27,32]. 

**Figure 8:**
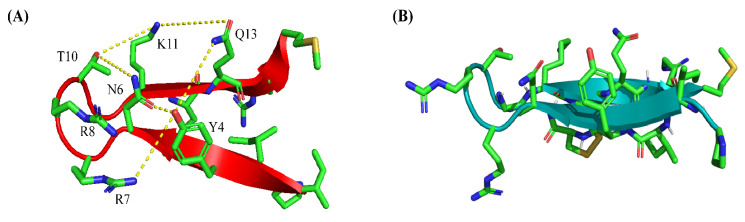
Comparison of sidechain packing and interactions within the β-hairpin structures of PM15 peptide in free solution and in complex with LPS micelle. (**A**) A network of polar interactions involving sidechains of residues Y4, R7, R8, N6, T10, K11 and Q13 on one face of the β-hairpin structure of PM15 in complex with LPS micelle. (**B**) β-hairpin structure of PM15 at the same orientation in free solution is shown for comparison.

**Figure 9:**
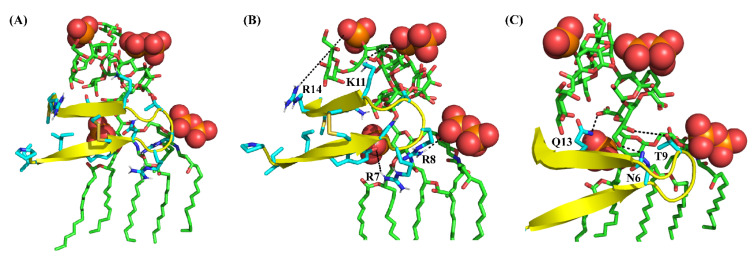
Docked complex, obtained by AutoDock, of LPS and PM15 revealing potential molecular interactions. (**A**) The overall topology of the complex of LPS and the β-hairpin structure of PM15 obtained in complex of LPS micelle. (**B**) Potential salt-bridge or ionic interactions between the sidechains of residues R7 and R8 with the bis-phosphate groups of lipid A moiety of LPS. Ionic interactions are probable, involving sidechains of residues K11 and R14 with phosphate groups of KDO sugar. (**C**) Polar contacts involving sidechains of residues N6, T9 and Q13 with the sugar moieties of LPS.

Atomic-resolution structures of PM15 and PM15Y peptides in complex with LPS micelle provide plausible mechanistic insights into the OM permeabilization process. Observations of more NOE contacts, including long-range NOEs, compared to free solution indicated that PM15 and PM15Y peptides experienced conformational changes in complex with the LPS–outer membrane. In free solution and in complex with LPS micelle, the β-hairpin structures of the peptides are deduced; however, significant differences in terms of sidechain orientations and backbone topology are revealed (Figure 8). In complex of LPS, a network of polar interactions is stabilized among sidechains of residues Y4, N6, R7, R8, T10, K11 and Q13 (Figure 8A). By contrast, the free structure of PM15 appears to be lacking these interactions (Figure 8B). Further, a docked complex of LPS/PM15 shows potential orientation within the LPS micelle [49] and outer-membrane interactions (Figure 9A). In the docked complex, the β-hairpin structure of LPS-bound PM15 assumes a parallel orientation on the surface of the LPS–outer membrane that can be stabilized by predominantly polar interactions (Figure 9B). In particular, potential salt bridges or ionic interactions are viable between the anionic phosphate groups of the lipid A of LPS with the cationic sidechains of residues R7 and R8 (Figure 9B). More ionic interactions are plausible involving the sidechains of residues K11 and R14 of PM15 with negatively charged phosphates of sugar residues of LPS (Figure 9B). In addition, the sidechains of residues N6, T9 and Q13 are in proximity to the hydroxyl groups of KDO sugars of LPS (Figure 9C). 

Notably, ITC and zeta potential studies indicated that polar/ionic interactions are prevalent in stabilizing binding of the thanatin-derived peptides with the LPS–outer membrane. Mechanistically, the mode of binding of PM15 and analog peptides with the LPS head groups (phosphates and sugars) may cause displacement of the OM-stabilizing divalent cations with concomitant reduction in acyl chain packing among LPS molecules. The process would essentially favor translocation of the peptides to the bacterial periplasmic space through a permeabilized OM. Binding of thanatin to LptA has been recognized as an important molecular event that causes growth inhibition of Gram-negative bacteria. Due to oligomerization of full-length LptA, a truncated but functional variant, LptA_m_, is often utilized for structural and biophysical studies with thanatin [33,50]. PM15 and PM15Y bind to LptA_m_ with high affinity, suggesting their ability to inhibit LPS transport to the outer membrane. In conclusion, this study has elucidated antibacterial activity and mode of action of peptide fragments of thanatin, a potent AMP against drug-resistant Gram-negative pathogen. The short active AMPs derived from thanatin are used to provide valuable lead molecules for the design and development of much-needed antibiotics to fight infections caused by MDR bacteria.

## 4. Materials and Methods

### 4.1. Peptides, Bacterial Strains and Media 

Chemically synthesized PM15, PM15Y and PM15A peptides were obtained from GLBiochem^TM^ (Shanghai, China). Molecular weight of the peptides was confirmed by mass spectrometry. Bacterial strains were obtained from the American Type Culture Collection (ATCC, Manassas, VA, USA). Mueller–Hinton (MH) broth was purchased from Sigma-Aldrich^TM^ (Saint Louis, MO, USA) and Millipore-Sigma^TM^ (Saint Louis, MO, USA). 1-N-phenylnaphthylamine (NPN) and LPS (*E. coli* O111:B4) were purchased from Sigma Aldrich^TM^. 

### 4.2. Determination of Minimal Inhibitory Concentration (MIC) of the Peptides

Bacterial growth inhibition of PM15, PM15A and PM15Y peptides was estimated against a panel of bacterial strains, namely, *E. coli* ATCC25922, *S. enterica* ATCC 14028, *K. pneumoniae* ATCC 13883, *A. baumannii* ATCC BAA-1798, *S. pyogenes* ATCC 19615 and *E. faecalis* ATCC 29212, by using the broth dilution assay determining minimum inhibitory concentration (MIC). Typically, overnight bacterial cultures were grown to mid-log phase at 37 °C and then diluted in Mueller–Hinton (MH) broth to OD_600_ ~ 0.002. In a 96-well plate, bacterial culture, final cell density 10^5^ CFU/mL and peptide of graded concentrations (0 μM to 16 μM) were incubated overnight at 37 °C. Wells for positive and negative controls contained bacteria and MH broth, respectively. Bacterial inhibition was determined by absorbance of 96-well plates at OD_600_ nm by using a Cytation spectrophotometer (Agilent, Santa Clara, CA, USA). The minimum peptide concentration showing complete inhibition of bacterial growth was determined as the MIC value. 

### 4.3. NPN Fluorescence Assay 

Peptides induced in NPN fluorescence experiments were measured to define the extent of outer-membrane permeabilization of *E. coli* cells. NPN assay was carried out in a Cary Eclipse fluorescence spectrophotometer (Varian, Palo Alto, CA, USA) by using a quartz cuvette of 0.1 cm path-length. Mid-log-phase *E. coli* cells in LB media were centrifuged at 5000 rpm for 10 min and resuspended in 10 mM sodium phosphate buffer at an OD_600_~0.5. NPN (10 µM) fluorescence, excitation 350 nm, emission 390–450 nm, in bacterial cell suspension was obtained in the absence of peptide and in the presence of various concentrations, ranging from 1–16 µM, of peptides. The results were expressed as ΔF vs. peptide concentration in which the basal fluorescence was subtracted from the maximum intensity recorded for each peptide concentration.

### 4.4. Zeta Potential Measurements 

All zeta potential measurements were performed on a Zeta Sizer Nano ZS (Malvern Instruments, Worcestershire, UK) equipped with a 633 nm He laser using zeta cells with gold electrodes. Typically, *E. coli* cells at a mid-log culture in LB media were harvested by centrifugation at 5000 rpm for 10 min and resuspended in 10 mM sodium phosphate buffer, pH 7. Zeta potential value of the bacterial cell suspension was determined for the free cells and with subsequent additions of peptide (2 μM to 32 μM). For each concentration, 100 runs were carried out and three replicates were executed. 

### 4.5. Isothermal Titration Calorimetry (ITC) Studies 

ITC experiments were conducted by using a MicroCal ITC 200 calorimeter. Samples, LPS and peptides, were prepared in 10 mM sodium phosphate buffer, pH 7.0. LPS (50 µM) was placed in the sample cell, while the syringe contained 1 mM of peptide. In an individual experiment, 2.0 μL of peptide was titrated, with a total of 20 titrations, into the sample cell containing LPS at 37 °C with stirring speed adjusted to 750 rpm. For ITCs of peptides/LptA_m_, samples were prepared in 50 mM sodium phosphate buffer, 150 mM NaCl, pH 7.0. 25 μM of LptA_m_ was loaded into the sample cell, whereas 250 μM of PM15 and PM15Y was placed in the syringe. ITC titrations were performed with 20 injections of 2 µL of peptide into sample cells at 25 °C with a stirring speed of 750 rpm. ITC data were analyzed by using a single site binding model in MicroCal Origin 5.0 software to obtain the association constant (K_a_) and enthalpy change (ΔH). Other binding parameters, namely, dissociation constant (K_d_), Gibbs free energy (ΔG) and entropy change (TΔS), were determined indirectly from thermodynamics equations. 

### 4.6. Purification of LptA_m_ protein

Expression and purification of LptA_m_ protein (residues 28–159), with a six His-tag (SGRVEHHHHHH) at the C-terminus, was carried out by using a previously established method [27]. Briefly, plasmid DNA coding for LptA_m_ gene was transformed into *E. coli* BL21 competent cells in LB agar plates containing 100 µg/mL of ampicillin. Protein production from expressing *E. coli* was induced by 1 mM isopropyl β-d-1-thiogalactopyranoside (IPTG) at an OD_600_ value of 0.6–0.8 at 18 °C for 18 h. The bacteria cells were centrifuged at 6000 rpm, 4 °C for 15 min and resuspended in a lysis buffer (100 mM HEPES, 500 mM NaCl, 10 mM imidazole, pH 8). Bacterial cells were lysed by sonication on ice at 25 Amp for 1 h, the lysate was centrifuged at 18,000 rpm, 4 °C for 30 min to remove cellular debris. The cell supernatant was applied through Ni-NTA (Qiagen, Germantown, TN, USA) beads thrice before washing it with increasing concentrations of imidazole. Bound his-tagged LptA_m_ was eluted from the beads with high imidazole buffer (20 mM HEPES, 150 mM NaCl, 200 mM imidazole, pH 8). Protein was further purified by size-exclusion chromatography using buffer (50 mM sodium phosphate buffer, 150 mM NaCl, pH, 7). 

### 4.7. NMR Experiments, Structure Determination of PM15, PM15Y Peptides and LPS-PM15 Docking

All NMR studies of PM15 and PM15Y were recorded on a Bruker DRX 600 spectrometer, equipped with a cryo-probe and pulse field gradients. NMR data were processed and analyzed by using Topspin and SPARKY (T.D. Goddard and D.G. Kneller, University of California, San Francisco, CA, USA). NMR samples of peptides, concentration 300 μM, were prepared in aqueous solutions containing 10% D_2_O at pH 5. Two-dimensional ^1^H-^1^H TOCSY (total correlation spectroscopy) and NOESY (nuclear Overhauser effect spectroscopy) spectra were acquired at 278 K. Two-dimensional ^1^H-^1^H tr-NOESY experiments were conducted for both peptides in the presence of 20 μM LPS micellooe. Chemical shift was directly referenced to DSS (2,2-dimethyl-2-silapentane 5-sulfonate sodium salt) at 0 ppm. Three-dimensional structures of free peptides and in complex with LPS micelle were determined by using the CYANA program [43]. Upper bounds of the distance constraints, 2.5, 3.5 and 5.0 Å, were obtained from NOESY peak intensity and were classified as strong, medium and weak NOEs. PREDITOR [42] was used to estimate backbone dihedral angles (ϕ and φ) using Hα chemical shift deviation of individual amino acids. Out of 100 structures calculated, 20 structures with the lowest energy were selected to represent the ensemble and used for further analysis. The stereochemical quality of the structural ensembles was assessed from PROCHECK107 analyses. PM15 and LPS complex model was derived by using the AutoDock Vina tool [51]. The LPS and PM15 molecules were prepared by using the prepare_ligand tool from the ADFR package. 

## Figures and Tables

**Table 1:** Minimum inhibitory concentrations (MIC) values (in µM) of PM15, PM15Y and PM15A peptides against Gram-negative (EC: *Escherichia coli* ATCC 25922, KP: *Klebsiella pneumoniae* ATCC 13883, SE: *Salmonella enterica* ATCC 14028, AB: *Acenobacter baumannii* ATCC BAA-1798) and Gram-positive bacteria (SP: *Streptococcus pyogen* ATCC 19615 and EF: *Enterococcus faecalis* ATCC 29212) in an aged MH broth (Sigma) and in new MH broth (Millipore); MIC values observed in new MH broth (Millipore) are given in paratheses.

Peptides	Gram-Negative				Gram-Positive	
	EC	KP	SE	AB	SP	EF
**PM15**	4 (>16)	4 (>16)	4–8 (>16)	>16 (>16)	8 (>16)	8 (>16)
**PM15A**	8 (>16)	4 (>16)	8 (>16)	>16 (>16)	8 (>16)	8 (>16)
**PM15Y**	4 (>16)	4 (>16)	>16(>16)	>16 (>16)	8 (>16)	8(>16)

**Table 2:** Binding interactions and thermodynamic parameters of PM15, PM15A and PM15Y peptides with LPS in sodium phosphate buffer, pH 7.

Peptides	K_d_ (μM)	ΔH (kcal/mol)	TΔS (kcal/mol)	ΔG (kcal/mol)
**PM15**	0.57	−7.33	1.54	−8.87
**PM15A**	0.33	−8.74	0.47	−9.21
**PM15Y**	0.28	−11.79	−2.49	−9.30

**Table 3:** Long-range NOEs observed in ^1^H-^1^H NOESY spectra of PM15 in aqueous solution and in complex with LPS micelle.

Free PM15	PM15 in LPS Micelle
2 ILE ^δ^H_3_–14 ARG ^ε^H 3 ILE ^δ^H_3_–13 GLN H 5 CYS ^α^H–13 GLN H 5 CYS ^α^H–12 CYS ^α^H 5 CYS ^α^H–12 CYS H 6 ASN H–11 LYS H 6 ASN H–13 GLN H 6 ASN ^β^H2–11 LYS H 6 ASN ^β^H3–11 LYS H 12 CYS ^α^H–6 ASN H 12 CYS ^β^H–5 CYS ^α^H 13 GLN ^β^Hs–4 TYR ^δ^Hs 13 GLN ^γ^Hs–4 TYR ^δ^Hs	2 ILE ^δ^H_3_–14 ARG ^ε^H 3 ILE ^δ^H_3_–13 GLN H 4 TYR H–13 GLN H 5 CYS ^α^H–13 GLN H 5 CYS ^α^H–12 CYS H 5 CYS ^β^H_2_–12 CYS H 6 ASN H–11 LYS H 6 ASN H–13 GLN H 6 ASN ^β^H2–11 LYS H 6 ASN ^β^H3–11 LYS H 11 LYS H–6 ASN ^δ^Hs 11 LYS ^β^Hs–6 ASN H 12 CYS ^α^H–6 ASN H 12 CYS ^β^Hs–5 CYS H 12 CYS ^β^Hs–5 CYS ^α^H 13 GLN ^β^Hs–4 TYR ^δ^Hs 13 GLN ^γ^Hs–4 TYR ^δ^Hs 13 GLN ^γ^Hs–4 TYR ^ε^Hs

**Table 4:** Long-range NOEs observed in ^1^H-^1^H NOESY spectra of PM15Y in aqueous solution and in complex with LPS micelle.

Free PM15Y	PM15Y in LPS Micelle
3 ILE ^α^H–14 ARG H 3 ILE ^α^H–15 MET H 3 ILE ^δ^H_3_–13 GLN ^α^H 5 CYS ^α^H–13 GLN H 6 ASN ^β^H_2_–11 LYS H 6 ASN ^β^H_3_–11 LYS H 13 CYS ^α^H–6 ASN ^α^H 13 GLN ^γ^H_2_–4 TYR ^δ^H_s_ 15 MET ^γ^H_2_–1 TYR ^δ^H_s_ 15 MET ^β^H_2_–1 TYR ^δ^H_s_	3 ILE ^α^H–14 ARG H 3 ILE ^α^H–15 MET H 2 ILE ^γ^H_2_–14 ARG H 3 ILE ^δ^H_3_–13 GLN H 3 ILE ^δ^H_3_–15 MET H 4 ILE ^δ^H_3_–13 GLN H 4 TYR H–13 GLN H 4 TYR ^α^H–13 GLN H 5 CYS H–13 GLN H 5 CYS ^α^H–13 GLN H 6 ASN H–11 LYS H 6 ASN ^β^H_2_–11 LYS H 6 ASN ^β^H_3_–11 LYS H 12 CYS ^α^H–6 ASN ^α^H 13 GLN ^γ^H_2_–4 TYR ^δ^H_s_ 13 GLN ^γ^H_3_–4 TYR ^δ^H_s_ 15 MET ^β^H_2_–1 TYR ^δ^H_s_ 15 MET ^β^H_3_–1 TYR ^δ^H_s_ 15 MET ^β^H_2_–1 TYR ^Ɛ^H_s_ 15 MET ^γ^H_2_–1 TYR ^Ɛ^H_s_ 15 MET ^γ^H_2_–4 TYR ^δ^H_s_

**Table 5:** Structural statistics of PM15 and PM15Y peptides in aqueous solution and in complex of LPS micelles.

	Free PM15	PM15 in LPS	Free PM15Y	PM15Y in LPS
**Distance constraints**				
Intra-residue [|i − j| = 0]	67	73	87	69
Sequential [|i − j| = 1]	34	50	32	45
Medium range [1 < |i − j| < 4]	11	12	5	12
Long range [|i − j| ≥ 4]	13	18	10	21
Total NOE	128	156	134	147
**Dihedral angle constraints (φ, ψ)**	24	24	24	24
**Deviation from mean structure**				
All backbone atoms (Å)	0.58	0.44	0.40	0.55
All heavy atoms (Å)	1.51	1.27	1.14	1.45
**Ramachandran plot for the mean structure**				
% of residues in most favored region and additional allowed region	100	100	100	100
% of residues in generously allowed region	0	0	0	0
% of residues in disallowed region	0	0	0	0

**Table 6:** Thermodynamic parameters of interaction between PM15 and PM15Y with LptA_m_ in 50 mM sodium phosphate and 150 mM NaCl buffer, pH 7.

	K_d_ (μM)	ΔH (Kcal/mol)	TΔS (Kcal/mol)	ΔG (Kcal/mol)
**PM15**	0.60	−2.93	6.23	−9.16
**PM15Y**	0.40	−11.79	−2.49	−9.30

## Data Availability

Data are contained within the article.
